# Effects of Three Types of Japanese Honey on Full-Thickness Wound in Mice

**DOI:** 10.1155/2013/504537

**Published:** 2013-01-20

**Authors:** Yukari Nakajima, Yuki Nakano, Sono Fuwano, Natsumi Hayashi, Yukiho Hiratoko, Ayaka Kinoshita, Megumi Miyahara, Tsuyoshi Mochizuki, Kasumi Nishino, Yusuke Tsuruhara, Yoshika Yokokawa, Terumi Iuchi, Yuka Kon, Kanae Mukai, Yukie Kitayama, Naoko Murakado, Mayumi Okuwa, Toshio Nakatani

**Affiliations:** ^1^Graduate Course of Nursing Science, Division of Health Sciences, Department of Clinical Nursing, Graduate School of Medical Science, Kanazawa University, 5-11-80 Kodatsuno, Kanazawa 920-0942, Japan; ^2^Department of Nursing, School of Health Sciences, Kanazawa University, 5-11-80 Kodatsuno, Kanazawa 920-0942, Japan

## Abstract

Although many previous studies reported that honey promotes wound healing, no study has examined the effects of Japanese honey. The aim of this study was to investigate the effects of three types of Japanese honey, Acacia, Buckwheat flour, and Chinese milk vetch honey, on wound healing in comparison with hydrocolloid dressing. Circular full-thickness skin wounds were produced on male mice. Japanese honey or hydrocolloid dressing was applied daily to the mice for 14 days. The ratio of wound area for the hydrocolloid dressing group increased initially in the inflammatory and early proliferative phases and then decreased rapidly to heal with scarring. However, the ratios of wound area for the Japanese honey groups decreased in the inflammatory phase, increased in the proliferative phase, and decreased in the proliferative phase, and some wounds were not completely covered with new epithelium. These findings indicate that using Japanese honey alone has limited benefit, but since it reduces wound size in the inflammatory phase, it is possible to apply a combined treatment in which Japanese honey is applied only in the inflammatory phase, followed by hydrocolloid dressing from the proliferative phase, which would effectively contract the wound.

## 1. Introduction

Wound healing is a dynamic physiological process initiated and influenced by many factors [1]. The process can be divided into four stages: hemostasis, inflammation, proliferation (the formation of granulation, contraction, and reepithelialization), and remodeling. In hemostasis, as the blood components enter the site of injury, the platelets release essential growth factors and cytokines such as platelet-derived growth factor (PDGF) and transforming growth factor beta (TGF-*β*). In the inflammatory phase, neutrophils enter the wound and begin the critical task of phagocytosis to remove foreign materials, bacteria, and damaged tissue. Macrophages appear and continue the process of phagocytosis as well as releasing more PDGF and TGF-*β*. Once the wound site is cleaned out, fibroblasts migrate in to begin the proliferative phase and deposit new extracellular matrix. Some fibroblasts may correspond to myofibroblasts, which are distributed along the wound edge and wound bed and cooperate in wound contraction [2]. Thereafter, myofibroblasts increase in number, and collagen fibers are produced as well as myofibroblasts. The new collagen matrix then becomes cross-linked and organized during the final remodeling phase [1].

Many studies on honey produced in countries besides Japan have been conducted, for example, Indonesia (Indonesian honey) [3], Turkey (chestnut honey, pure rhododendron honey, and pure blossom honey) [4], Malaysia (Gelam honey and Tualang honey) [5–8], Iran (Urmia honey) [9], Pakistan (Acacia honey) [10], and Nigeria (Jungle honey) [11]. In developing countries, honey has been used as a treatment for various wounds [12], while it is unfamiliar in Japan. Honey is reported to have a debriding effect [12, 13]; however, its mechanism of debriding action has not yet been explained [14], and it decreases infection because of its antibacterial activity: high osmotic effect, acidity, hydrogen peroxide, and phytochemical factors [15]. In addition, it also decreases inflammation [5, 16] and wound area [3, 5, 6]. The anti-inflammatory action of honey decreases edema [8] and the high osmotic pressure of honey dehydrates tissue edema [17]. Wound area reduction in the inflammatory phase results from anti-inflammatory properties [9, 12, 14] and antibacterial activity by the hydrogen peroxidase in honey [12, 18]. It also has a pH from 3 to 4, and topical acidification causes oxygen release from hemoglobin [14]; in addition, the hydrogen peroxide contained at low levels in honey also stimulates angiogenesis [5] and the growth of fibroblasts. Honey enhances wound contraction by stimulating fibroblasts, myofibroblasts, and collagen deposition by providing a source of energy, namely, sugar [3, 7, 19]. Moreover, it promotes reepithelialization [4]. Acceleration of reepithelialization results from its high osmotic pressure, which dehydrates tissue edema and holds the wound edges together [17], and by the presence of hydrogen peroxide, which stimulates the growth of epithelial cells [10]. Therefore, honey has positive effects on the wound healing process.

Honey is a natural product and its characteristics associated with wound healing may be affected by the species of bee, geographical location, and botanical origin, as well as processing and storage conditions [20]. In general, pure commercial unheated honey is composed of approximately 40% glucose, 40% fructose, 20% water, amino acids, the vitamin biotin, aminonicotinic acid, folic acid, pantothenic acid, pyridoxine, thiamine, the enzymes diastase invertase, glucose oxidase, and catalase, and the minerals calcium, iron, magnesium, phosphorus and potassium [21]; honey also contains bee pollen enzymes and propolis, all of which can stimulate new tissue growth; it may also contain other medicinal compounds, including essential oils, flavonoids, terpenes, and polyphenols, depending on the plant from which the pollen was taken [22, 23]. On the other hand, concerning the three types of honey in this study, which are produced in Japan and familiar to the Japanese, Acacia honey is composed of 70.8% glucose and fructose and 18.6% water, Buckwheat flour honey is composed of 71.2% glucose and fructose and 17.2% water, and Chinese milk vetch honey is composed of 71.0% glucose and fructose and 18.4% water (Yamada Bee Farm, Okayama, Japan). Although more detailed information on their compositions is not known, since they contain a lot of sugar and water like honey produced in other countries, they seem to have the same effects on wound healing. However, to our knowledge, in Japan, there have been no studies evaluating the use of Japanese honey as a topical therapy in wound care both macroscopically and microscopically, so it is very important to clarify the effect of Japanese honey on wound healing. If we identify that Japanese types of honey have the same or better effects than hydrocolloid dressing or honey from other countries, we can use Japanese honey as an alternative dressing for wound care; such treatment using honey will enable cost reductions for both patients and institutions. 

Against this background, we hypothesize that Japanese honey also promotes wound healing as well as honey from other countries; that is, it decreases inflammation and wound area, increases reepithelialization, contraction, and deposition of collagen, and promotes overall wound healing. Therefore, the aim of this study is to clarify the effects of Japanese types of honey on the wound healing process.

## 2. Materials and Methods

### 2.1. Animals

Seventy-two BALB/cCrSlc male mice aged 8 weeks (Sankyo Lab Service Corporation, Inc., Toyama, Japan) and weighing 21.3–26.0 g were used. They were caged individually in an air-conditioned room a t 25.0 ± 2.0°C with light from 08:45 to 20:45 hours. Water and laboratory chow were given freely. The experimental protocol and animal care were in accordance with the Guidelines for the Care and Use of Laboratory Animals of Kanazawa University, Japan (AP-112200).

### 2.2. Honey

Three types of honey were used: Acacia (*Robinia pseudoacacia*), Buckwheat flour (*Fagopyrum esculentum*) honey, and Chinese milk vetch (*Astragalus sinicus*) honey(Yamada Bee Farm, Okayama, Japan).

### 2.3. Injury Induction

In accordance with previous studies [3, 24, 25], the mice were anesthetized with an intraperitoneal (IP) injection of pentobarbital sodium (0.05 mg/g weight), and the dorsum was shaved. Two circular (4 mm in diameter) full-thickness skin wounds including the panniculus muscle on both sides of the dorsum of the mouse were made with a Kai sterile disposable biopsy punch (Kai Industries, Gifu, Japan). We chose two circular wounds on each mouse because this method decreases the number of mice required, as shown in our previous studies. Mice were divided into four groups (Table 1). Wounds of the experimental groups, Acacia honey, Buckwheat flour honey, and Chinese milk vetch honey groups, were treated with 0.1 mL of honey per wound. The wounds to which honey was applied were covered with gauze to prevent the honey from running off, and the mice were wrapped twice with a sticky bandage (Mesh pore tape; Nichiban, Tokyo, Japan). The gauze was changed and all wounds were treated with honey every day. Meanwhile, wounds of the control group were covered with hydrocolloid dressing (Tegaderm; 3 M Health Care, Tokyo, Japan) to maintain a moist environment. All control mice were wrapped twice with sticky bandages, the same as the experimental groups.

### 2.4. Macroscopic Observation

The day when wounds were made was designated as day 0, and the process of wound healing was observed from day 0 to 14 after wounding. We observed edema, infection, and necrotic tissue on each wound. Wounded edges were traced on polypropylene sheets, and photographs were taken every day. The traces on the sheets were captured with a scanner onto a personal computer using Adobe Photoshop Elements 7.0 (Adobe System Inc., Tokyo, Japan), and the areas of wounds were calculated using image analysis software Scion Image Beta 4.02 (Scion Corporation, Frederick, Maryland, USA).

### 2.5. Tissue Processing

The mice were euthanized by a massive pentobarbital sodium (0.5 mg/g weight) IP injection on days 3, 7, 11, and 14 after wounding. The wounds and the surrounding intact skin were harvested, stapled onto transparent plastic sheets to prevent overcontraction of specimens, and fixed in 4% paraformaldehyde in 0.2 mol/L phosphate buffer (pH 7.4) for 15 hours. Specimens were dehydrated in an alcohol series, cleaned in xylene, and embedded in paraffin to prepare 5 *μ*m serial sections. Sections of 5 *μ*m thickness were stained with hematoxylin-eosin (H-E) or subjected to Azan staining and immunohistologically stained with anti-neutrophil antibody (Abcam Japan, Tokyo, Japan) for detecting neutrophils, anti-mouse Mac-3 antibody (BD Pharmingen, Tokyo, Japan) for detecting macrophages, or anti-*α*-smooth muscle actin (*α*-SMA) antibody, prediluted (Abcam KK, Tokyo, Japan), for detecting myofibroblasts. The procedure for unmasking antigens was antigen-dependent, as detailed below.

### 2.6. Immunohistochemical Staining

After deparaffinization and rehydration, antigen unmasking was accomplished by heating slides in a water bath followed by incubation in sodium citrate buffer (10 mM sodium citrate, 0.05% Tween 20, pH 6.0) for 20 minutes at approximately 100°C. Slides for anti-mouse Mac-3 antibody and anti-*α*-SMA were washed with phosphate-buffered saline (PBS), and slides for anti-neutrophil antibody were washed with 0.3% Triton X-100 in PBS. Then, slides were incubated with anti-neutrophil antibody or Mac-3 antibody at a concentration of 1 : 100 in PBS or anti-*α*-SMA at 4°C overnight. Slides were again washed with PBS or 0.3% Triton X-100 in PBS. For detection of primary antibodies, slides for anti-mouse Mac-3 antibody and anti-neutrophil antibody were incubated with polyclonal rabbit anti-rat immunoglobulins/HRP (Dako North America, California, USA) at a concentration of 1 : 300 in 0.3% mouse serum (normal) (Dako North America, California, USA) in PBS for 30 minutes at 4°C, and slides for anti-*α*-SMA antibody were incubated with Dako Envision+ system-HRP labeled polymer anti-rabbit (ready to use) (Dako North America, California, USA) for 30 minutes at room temperature. Slides were again washed with PBS or 0.3% Triton X-100 in PBS and then incubated in Dako Liquid DAB+ Substrate Chromogen System (Dako North America, California, USA) (brown chromogen) for 5 minutes or until staining was detected at room temperature. Light hematoxylin counterstaining was applied for 1 minute for visualization of cell nuclei. Finally, slides were rinsed in distilled water, dehydrated, cleared, and mounted for analysis. Negative control slides were obtained by omitting each primary antibody.

### 2.7. Microscopic Observations

We measured the ratio of reepithelialization (%) = length of new epithelium/length of wound between wound edges, counted the number of neutrophils, macrophages, myofibroblasts, and blood vessels by observation through a light microscope with 400x magnification, and then calculated the ratio of each parameter/mm^2^ granulation tissue. The ratio of collagen fibers in granulation tissue = number of pixels of collagen fibers/number of pixels of granulation tissue area using Adobe Photoshop Element 7.0.

### 2.8. Statistical Analysis

Data are expressed as mean ± SD, analyzed using JMP 8.0.1 (SAS, USA) (ANOVA, multiple comparison Tukey-Kramer). The differences were considered significant at *P* < 0.05. 

## 3. Results

### 3.1. Macroscopic Observation of Wound Healing

**Figure 1:**
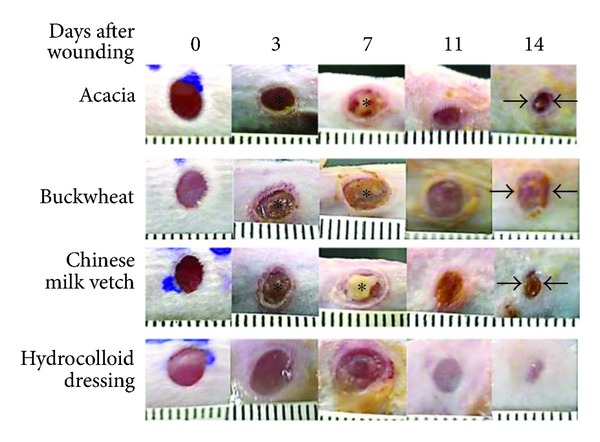
The process of wound healing in each group. Note the necrotic tissue (*) covering wound surfaces on day 7 and wound edges (arrows) on day 14 in the Japanese honey groups. On day 3, wound in the hydrocolloid dressing group is covered with a lot of exudate. The rulers indicate gradations of 1 millimeter.

When we treated each wound with honey every day, honey remained on the wound surfaces, wounds were moist, and each gauze with honey covering wounds was easily removed. Applied honey was viscid the next day, which could have been due to its absorption of exudate from the wound, and some of the honey leaked from the bandage covering the wound. On the other hand, hydrocolloid dressing absorbed so much of the exudate that it expanded, so the exudate did not spread out from the hydrocolloid. On days 3 to 5 after wounding, necrotic tissue clearly appeared on the surfaces of wound areas in the Japanese honey groups, and it covered the entirety of wounds on day 7, while necrotic tissue was not observed on wounds in the hydrocolloid dressing group (Figure 1). The wound in the hydrocolloid dressing group was clearly covered with the deposition of exudate until day 5 or 6. The exudate of the hydrocolloid dressing group decreased around day 7, while that of the Japanese honey groups was difficult to observe because honey, like exudate, is liquid (Figure 1). Since a lot of exudate was absorbed by honey and hydrocolloid dressing, it was unclear whether edema was present in the wound or the peripheral area of the wound in all groups. Signs of infection were not observed in any wounds.

**Figure 2:**
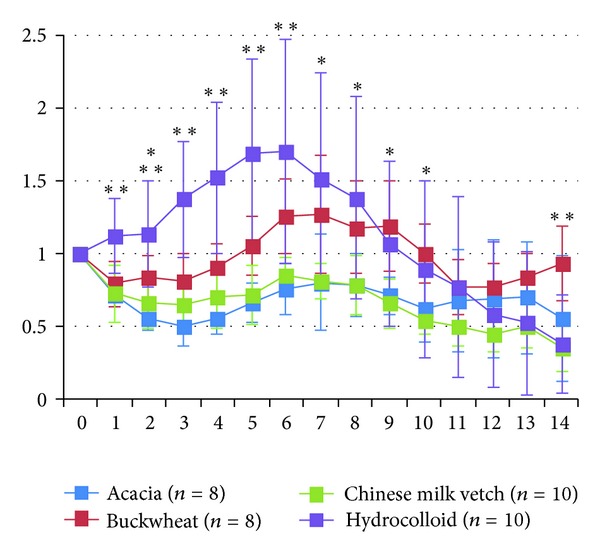
The ratios of wound areas to initial area on day 0 are shown on line graphs for each day. There were significant differences between the hydrocolloid dressing and Acacia honey, Buckwheat flour honey, and Chinese milk vetch honey groups on days 1 to 5 (*P* < 0.01). There were significant differences between the hydrocolloid dressing, Acacia honey, and Chinese milk vetch honey groups on days 6  (*P* < 0.01), 7, and 8 (*P* < 0.05). There were significant differences between the Buckwheat flour honey, and Acacia honey and Chinese milk vetch honey groups on day 9 (*P* < 0.05). There were significant differences between the Buckwheat flour honey and Chinese milk vetch honey groups on day 10  (*P* < 0.05). There were significant differences between the Buckwheat flour honey, Chinese milk vetch honey, and hydrocolloid dressing groups on day 14 (*P* < 0.01). Values are expressed as mean ± SD, ANOVA, Tukey-Kramer, *P < 0.05, **P < 0.01.

On days 1 to 14, the ratios of wound areas to the initial wound area on day 0 were calculated (Figure 2 and Table 2). In the hydrocolloid dressing group, the wound area increased gradually during the inflammatory and early proliferative phases, peaked on day 6, then more rapidly decreased during the proliferative phase, and wounds healed with scarring on day 14, being smaller in area than on day 0 (*P* < 0.0001). 

In the Acacia honey group, the wound area decreased gradually until day 3 during the inflammatory phase, being smaller in area than on day 0 (*P* = 0.0089), increased gradually until day 7 during the proliferative phase, decreased again gradually until day 10 during the proliferative phase, increased gradually until day 13, and then decreased on day 14 (day 0 versus day 14, *P* = 0.0400) during the remodeling phase. 

In the Buckwheat flour honey group, the wound area decreased on day 1, remained almost the same area until day 3 during the inflammatory phase, increased gradually until day 7 during the proliferative phase, being greater than the area on day 0, decreased gradually until day 12, and again increased until day 14 during the remodeling phase, being almost the same in terms of area as on day 0 (*P* = 1.0000). 

In the Chinese milk vetch honey group, the wound area decreased until day 3 during the inflammatory phase, being smaller in area than on day 0 (*P* = 0.0002), increased gradually until day 6 during the proliferative phase, and then decreased gradually until day 14 during the late proliferative and remodeling phases, being smaller in area than on day 0 (*P* = 0.0000). 

On day 14, when the wound of the hydrocolloid dressing group healed with scarring, there were no significant differences of wound area between the hydrocolloid dressing and the Acacia and Chinese milk vetch honey groups and between the Acacia and Chinese milk vetch honey groups, while there were significant differences between the Buckwheat flour honey and Chinese milk vetch honey groups (*P* = 0.0019) and the Buckwheat flour honey and hydrocolloid dressing groups (*P* = 0.0031) and a trend between the Acacia and Buckwheat flour honey groups (*P* = 0.0818). The wounds of the Acacia and Chinese milk vetch honey groups seemed to almost heal with red soft scarring like granulation tissue on day 14, and those of the Buckwheat flour honey group did not seem to heal with red large granulation tissue on day 14 (Figure 1).

### 3.2. Microscopic Observation

#### 3.2.1. Reepithelialization (Table 3 and Figures 3(a)–3(d))

Necrotic tissue covered almost all wound surfaces on days 3 and 7 in the Japanese honey groups as determined by macroscopic observation. On day 3 after wounding, the ratio of reepithelialization covering wound surface was almost the same between all groups. Thereafter, in only the hydrocolloid dressing group, new epithelium extended rapidly and covered about 77% of the wound surface until day 7 and then covered almost all of the wound surface on day 14. On the other hand, the ratio of new epithelium in the Japanese honey groups was much lower than that in the hydrocolloid dressing group on day 14 (*P* < 0.0001). The necrotic tissue covering the wound in the Japanese honey groups seemed to prevent new blood vessels. 

#### 3.2.2. New Blood Vessels (Table 3 and Figures 3(e)–3(h))

The number of new blood vessels per mm^2^ in the wound in the Japanese honey groups increased rapidly from day 3 to day 7 (each *P* < 0.0001) and then decreased gradually. In the hydrocolloid dressing group, it peaked on day 7 and then decreased rapidly to day 14. The numbers of blood vessels in the Japanese honey groups were larger than in the hydrocolloid dressing group on days 7, 11, and 14 (always *P* < 0.05). Since so many capillaries were observed in the granulation tissue in the Japanese honey groups on day 14, wounds did not seem to be scarring.

#### 3.2.3. Myofibroblasts (Table 4 and Figure 4)

Myofibroblasts had appeared in all wounds by day 3. The number of myofibroblasts per mm^2^ was almost the same in all groups. The number of myofibroblasts in the Japanese honey groups increased gradually from day 0 to day 14, while that in the hydrocolloid dressing group peaked on day 11 and after that decreased drastically on day 14. On day 7, the number of myofibroblasts in the hydrocolloid dressing group was larger than that of the Buckwheat flour honey and Chinese milk vetch honey groups (*P* = 0.0005, *P* < 0.0001, resp.), and the number of myofibroblasts in the Acacia honey group was larger than those of the Buckwheat flour honey and Chinese milk vetch honey groups (*P* = 0.0217, 0.0032, resp.).

#### 3.2.4. Collagen Fibers (Table 5 and Figure 5)

The ratio of collagen fibers stained with Azan stain in the wound increased gradually from day 3 to 14 in all groups. On day 7, the ratio of collagen fibers in the granulation tissue in the hydrocolloid dressing group was larger than those of the Acacia honey and Chinese milk vetch honey groups (*P* = 0.026, *P* = 0.005, resp.). On day 14, there was no significant difference between all groups, although the ratio of collagen fibers in the hydrocolloid dressing group seemed to be larger than in the Japanese honey groups.

#### 3.2.5. Macrophages (Table 6 and Figure 6)

Numerous macrophages were observed in the wound on day 3. On days 3 and 7 during the inflammatory and early proliferative phases, the number of macrophages per mm^2^ in the wound in the hydrocolloid dressing group was significantly larger than those in the Japanese honey groups (*P* < 0.0001). The number of macrophages in the hydrocolloid dressing group remained almost the same on days 3 and 7, and decreased gradually from day 7 to day 14 (*P* = 0.0091). However, those of the Japanese honey groups until day 14 remained almost the same as on day 3 (Acacia: *P* = 0.6665, Buckwheat flour: *P* = 0.9736, and Chinese milk vetch: *P* = 0.1317). 

#### 3.2.6. Neutrophils (Table 7 and Figure 7)

Numerous neutrophils appeared in the wound on day 3 at the inflammatory-phase, like macrophages. There were no significant differences in the number of neutrophils per mm^2^ in the wound between all groups on days 3 and 7; however, the Buckwheat flour honey group tended to have fewer neutrophils than the hydrocolloid dressing group (*P* = 0.0551) on day 3. On day 11, the Buckweat flour honey group had a larger number than the hydrocolloid dressing group (*P* = 0.0233), and the Buckweat flour honey group tended to have a large number than the hydrocolloid dressing group (*P* = 0.0697). On day 14, the number of neutrophils in the Chinese milk vetch honey group was larger than that of the hydrocolloid dressing group (*P* = 0.0478). The number of neutrophils in the hydrocolloid dressing group decreased rapidly from day 3 to day 14 (*P* = 0.0008), while the number of neutrophils in all Japanese honey groups until day 14 remained almost the same, from day 3 to day 14 (Acacia: *P* = 0.9635, Buckwheat flour: *P* = 0.9668, Chinese milk vetch: *P* = 0.9444). 

## 4. Discussion

Table 8 shows the differences between various types of honey in previous studies and Japanese honey in the present study. This shows that Japanese honey has some different effects from honey from other countries. 

The wound areas treated with the Acacia and Chinese milk vetch honey were almost the same as that of hydrocolloid dressing on day 14, so the Japanese honey may have an effect on wound healing, although the effect of Buckwheat honey on the wound healing was not clear. However, it is very difficult to explain clearly the phenomenon that the wound area treated with Japanese honey decreases during the inflammatory phase, increases during the proliferative phase, and then decreases during the remodeling phase. This may be due to the following phenomena observed in this study in the wounds treated with Japanese honey: the small number of macrophages that produce factors for wound healing [26]; the delay of production of myofibroblasts that contract the wound [27]; the retention of numerous neutrophils in the proliferation and remodeling phases, which appear at the inflammatory phase and thereafter decrease rapidly [3]; the small amount of deposition of collagen fibers in granulation tissue; the presence of numerous new blood vessels in granulation tissue on days 11 and 14, which appear in large quantities at granulation tissue and decrease rapidly late in the proliferation and remodeling phase [3, 4]. There are thus large differences between Japanese honey and that from other countries (Table 8). There is a need to clarify the reason for the differences between Japanese honey and that from other countries; both of which are composed of mainly water and sugar, as well as trace amounts of unknown, unique compounds that differ among each type of honey.

In the present study, wound areas treated with Japanese honey did not increase during the inflammatory phase, in comparison with hydrocolloid dressing, as well as in our previous study using Indonesian and Manuka honeys [3]. The effect of honey on the contraction of wound area or protection against enlargement of wound area has been reported in other studies [4, 10, 21]. Thus, contraction of wound area or suppression of the expansion of a wound during the inflammation phase is a very important effect of honey. It is likely that an increase of wound area in the inflammatory phase depends on the load for stretching, which pulls the wound edge by the breaking of collagen fibers, and the accumulation of exudate in the wound. Although hydrocolloid dressing is suitable to absorb wound exudate, which is produced at a high level during the inflammatory phase [25], the wound area covered with the hydrocolloid dressing increased; thus, it may not have such a good effect on inhibiting the production of exudate and the load for stretching of a wound. On the other hand, partly because honey has a high osmotic pressure produced by a high concentration of sugar, honey absorbs exudate like hydrocolloid dressing. In addition, partly because of anti-inflammatory action, which has been clarified by some reports [9, 12, 14] and recently by Hussein et al. [8], which inhibits the production of exudate, and partly because the viscosity of honey covering the wound may keep the wound edge together by resisting the stretching of collagen fibers, honey may contract the wound area or suppress expansion of the wound during the inflammatory phase. 

The retention of necrotic tissue on the wound surface for a long time and the prevention of extension of new epithelium on the wound surface were observed during the wound healing process in the Japanese honey groups. The former may have been due to the defect of debriding effect in Japanese honey, which is reported for honey from other countries [13, 20]. The latter may be due to the existence of necrotic tissue, which may physically prevent the migration of new epithelial cells, and the low number of macrophages at inflammatory and early proliferative phases, which secrete epidermal growth factor (EGF) that generates epidermis [28, 29]. 

These results indicate that the process of wound healing by Japanese honey was very different from that by honey from other countries. Therefore, we propose a combination treatment in which Japanese honey is applied only in the inflammatory phase, followed by hydrocolloid dressing from the proliferative phase, which produces effective contraction for wound healing. We will conduct a study on this issue next, and examine whether such treatment is effective to promote contraction and healing with less scar formation.

## 5. Conclusions

It was clarified that the process of wound healing by Japanese honey was very different from that by honey from other countries: there was specific wound area transition that decreased the wound area initially, then increased, and decreased it without complete reepithelialization. Therefore, it is suggested that Japanese types of honey should be applied only in the inflammatory phase to reduce wound area and should then be exchanged for hydrocolloid dressing from the proliferative phase to promote the formation of granulation tissue and collagen fibers.

## Figures and Tables

**Figure 3:**
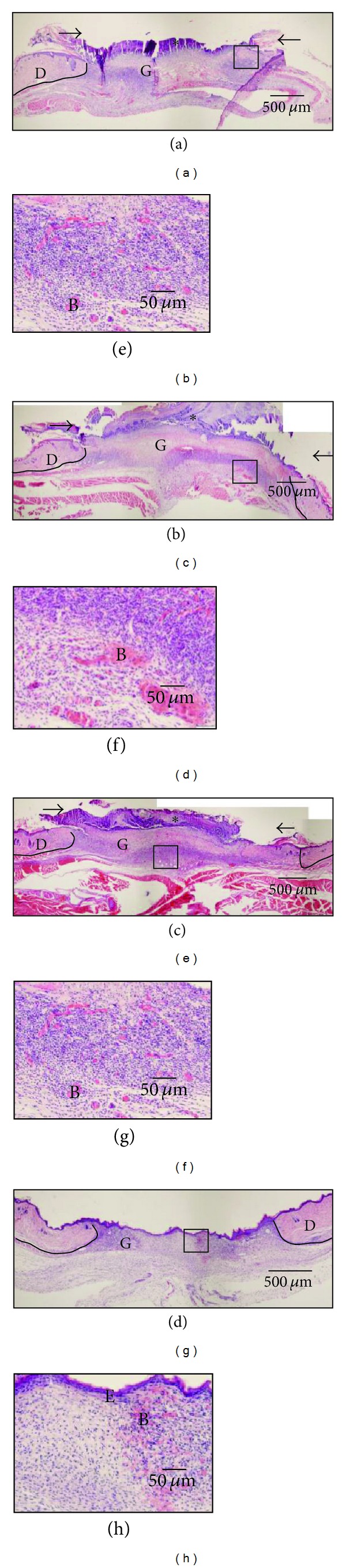
Japanese honey inhibits reepithelialization but increases vascularization. Note the necrotic tissue (*) covering wound surfaces and wound edges (arrows) in the Acacia honey (a), Buckwheat flour honey (b), and Chinese milk vetch honey (c) groups on day 7. This necrotic tissue appears to prevent the migration of epithelium on the wound surface. New epithelium is rapidly formed in the hydrocolloid dressing group (d). There are many large blood vessels in granulation tissue in the Japanese honey groups (e–g) compared with the case in the hydrocolloid dressing group (h). Squares in (a–d) are enlarged into (e–h). D: dermis, E: epidermis, G: granulation tissue, B: blood vessel. Solid line indicates the boundary between normal skin and wound.

**Figure 4:**
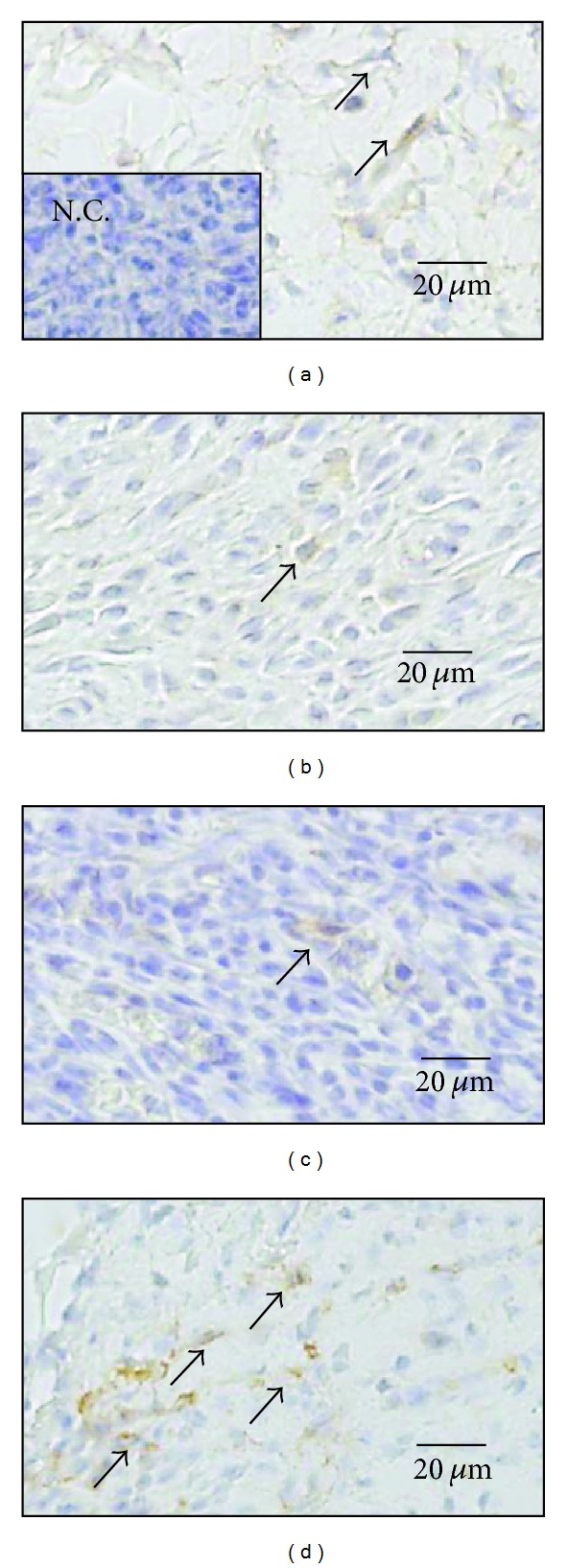
Myofibroblasts are present in wound. On day 7, myofibroblasts (arrows) stained with *α*-SMA antibody are observed in granulation tissue in the Acacia honey (a), Buckwheat flour honey (b), Chinese milk vetch honey (c), and hydrocolloid dressing (d) groups. They are elongated in shape. Negative control (N.C.) is inset in (a).

**Figure 5:**
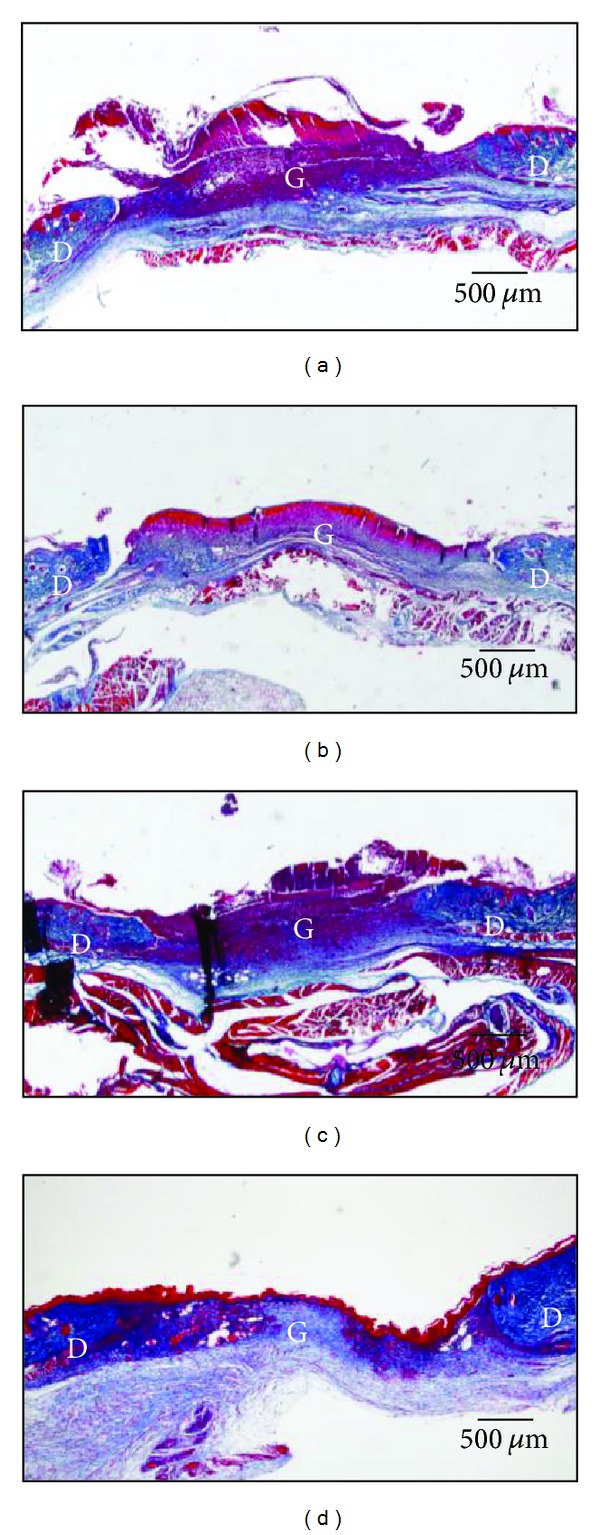
New collagen fibers are deposited in wound. On day 7, the ratio of collagen fibers stained with Azan staining colored in blue is observed in the granulation tissue (G) and dermis (D) in the Acacia honey (a), Buckwheat flour honey (b), Chinese milk vetch honey (c), and hydrocolloid dressing (d) groups. Necrotic tissue covering the granulation tissue is colored in red.

**Figure 6:**
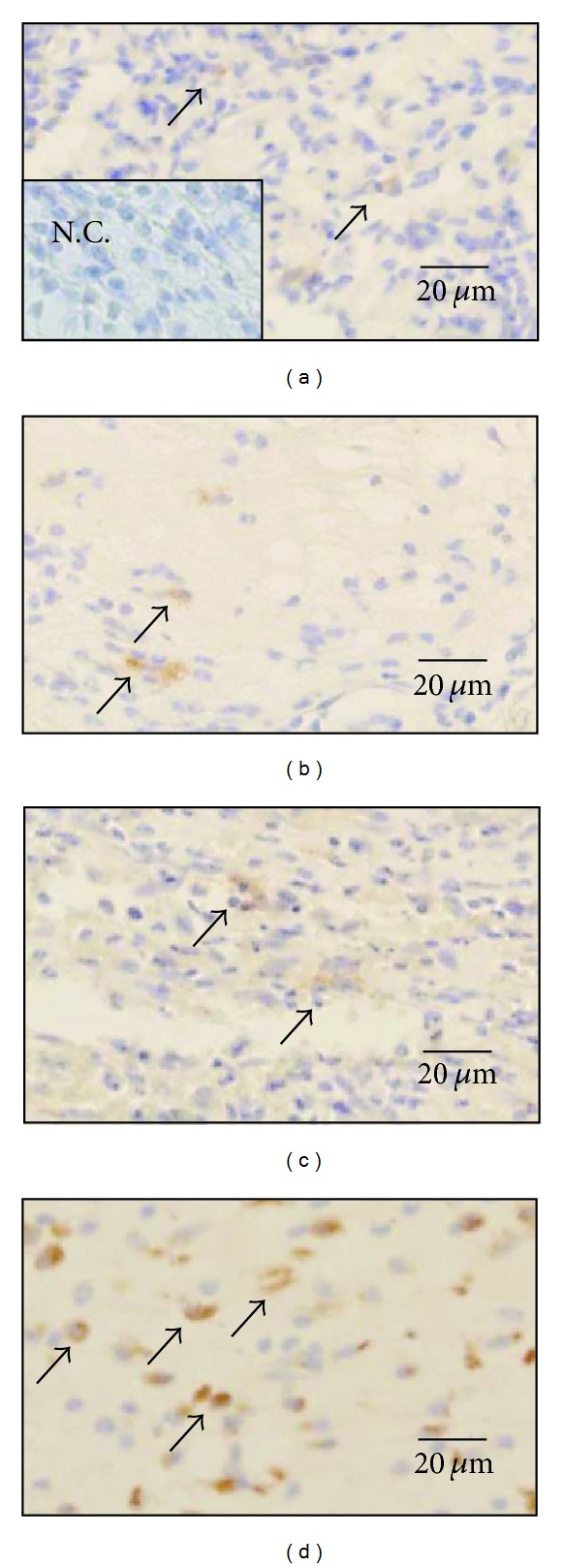
Macrophages are present in wound. On day 7, macrophages (arrows) stained with anti-mouse Mac-3 antibody are observed in the granulation tissue in the Acacia honey (a), Buckwheat flour honey (b), Chinese milk vetch honey (c), and hydrocolloid dressing (d) groups. Negative control (N.C.) is inset in (a).

**Figure 7:**
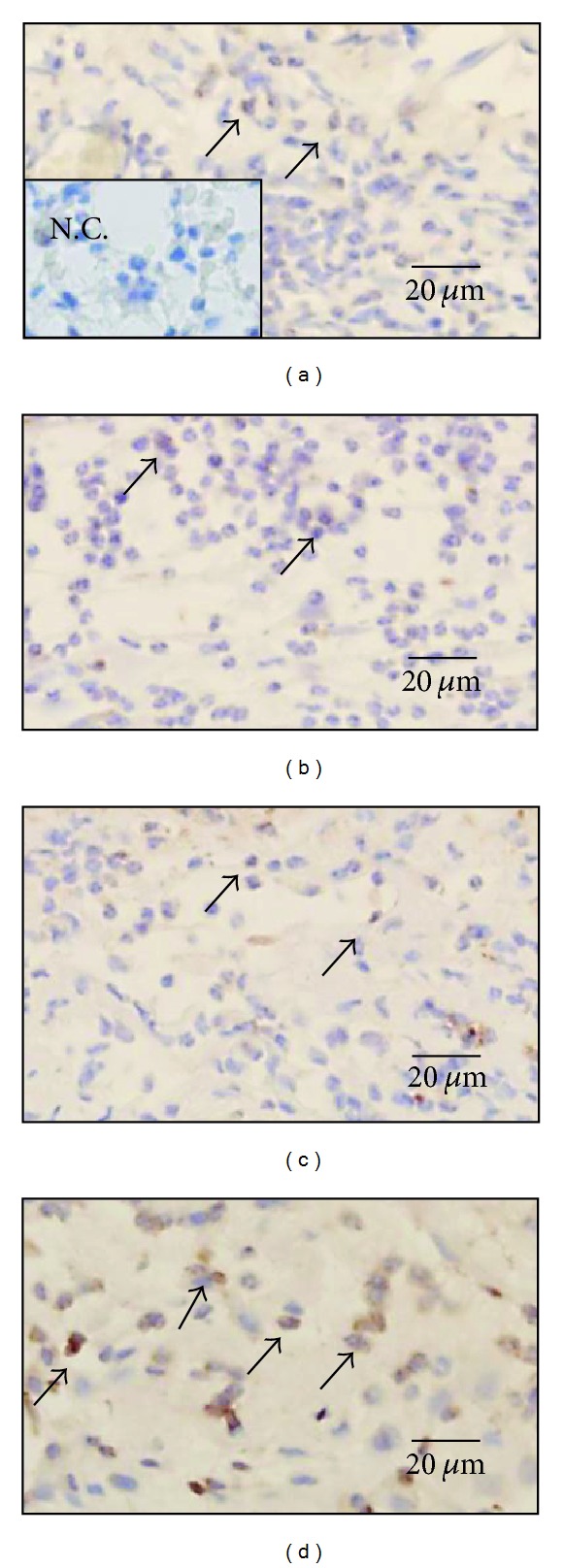
Neutrophils are present in wound. On day 3, neutrophils stained with anti-neutrophil antibody are observed in wound tissue at the inflammatory phase in the Acacia honey (a), Buckwheat flour honey (b), Chinese milk vetch honey (c), and hydrocolloid dressing (d) groups. Negative control (N.C.) is inset in (a). Neutrophils remain in the granulation tissue after the proliferative phase with the Japanese honey.

**Table 1:** The number of mice in each group and each day.

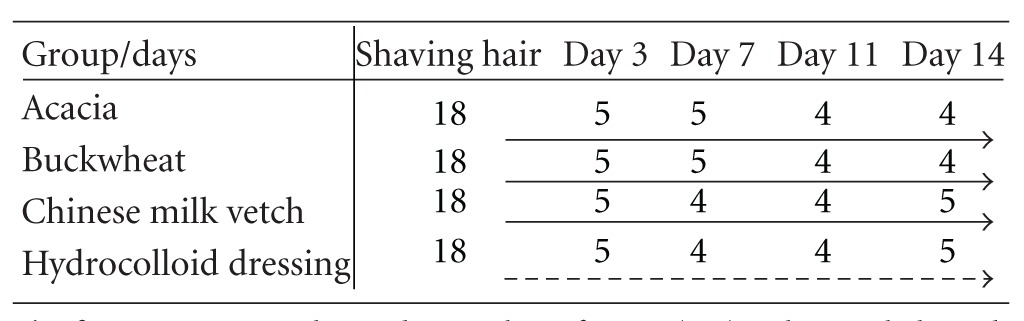

The figures 4, 5, 18 indicate the number of mice. (→) indicates daily each treatment with 0.1 mL of each honey per wound and covering with gauze and bandages. (⇢) indicates the daily treatment of covering with hydrocolloid dressing, gauze and bandage.

**Table 2:** The ratio of wound area to initial area on day 0.

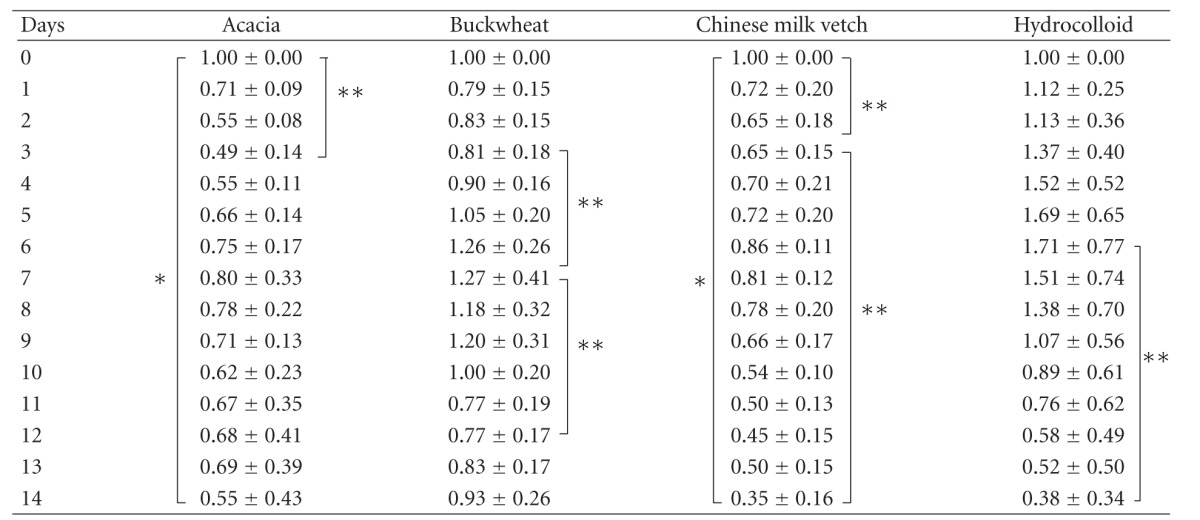

There are statistic significances between on days 0 and 14, and 3 in the Acacia group, and between on days 3 and 7 and between on days 7 and 12 in the Buckwheat flower honey group, and between on days 0 and 14, and 3, and between on days 3 and 14 in the Chinese milk vetch honey group, and between on days 6 and 14 in the hydrocolloid dressing group. Values are expressed as mean ± SD, ANOVA, Tukey-Kramer *P < 0.05 and **P < 0.01.

**Table 3:** The ratio of reepithelialization and the number of blood vessels in each group.

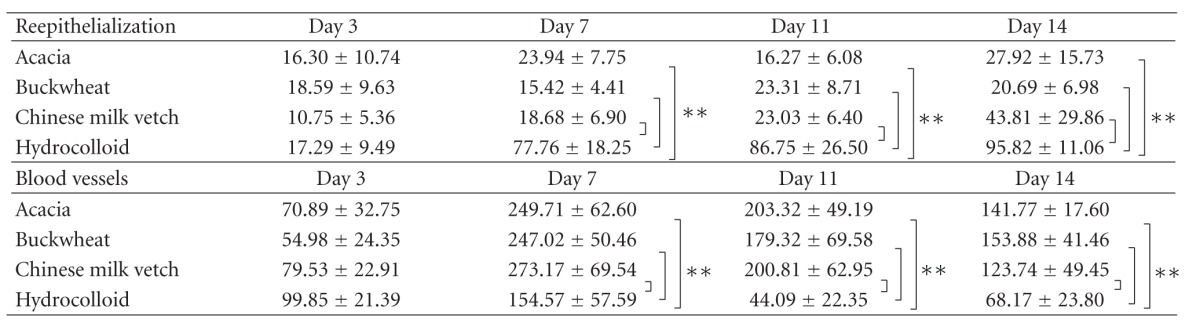

Rate of reepithelialization of wounds: *n* = 6-7 in the Acacia honey group, *n* = 5–8 in the Buckwheat flower honey group, *n* = 6–9 in the Chinese milk vetch honey group, and *n* = 4–8 in the hydrocolloid dressing group. The number of vessels of wounds: *n* = 5–7 in the Acacia honey group, *n* = 5–8 in the Buckwheat flower honey group, *n* = 6–9 in the Chinese milk vetch honey group, and *n* = 4–8 in the hydrocolloid dressing group. Values are expressed as mean ± SD, ANOVA, Tukey-Kramer **P < 0.01.

**Table 4:** The number of myofibroblasts in each group.

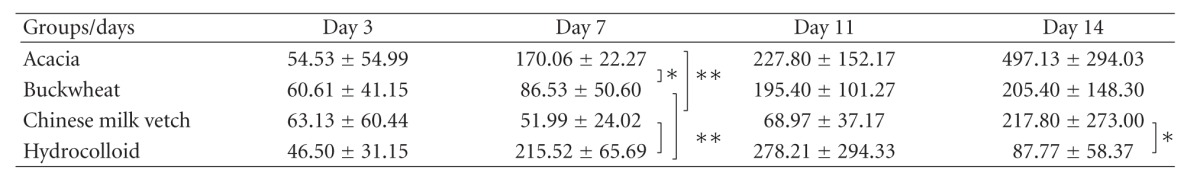

The number of myofibroblasts: *n* = 4-5 in the Acacia honey group, *n* = 4–8 in the Buckwheat flower honey group, *n* = 4–6 in the Chinese milk vetch honey group, and *n* = 4-5 in the hydrocolloid dressing group. Values are expressed as mean ± SD, ANOVA, Tukey-Kramer *P < 0.05, **P < 0.01.

**Table 5:** The ratio of collagen fibers in each group.

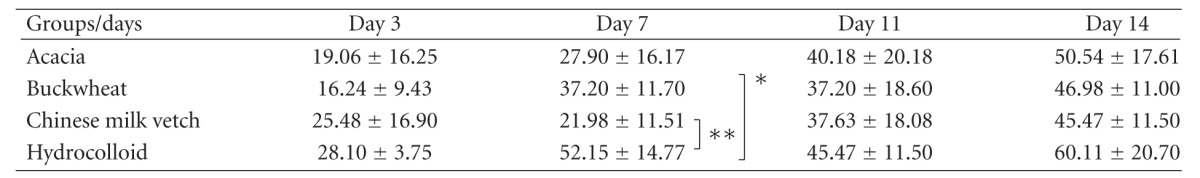

The rate of collagen fibers: *n* = 6-7 in the Acacia honey group, *n* = 5–7 in the Buckwheat flower honey group, *n* = 5–8 in the Chinese milk vetch honey group, and *n* = 5–7 in the hydrocolloid dressing group. Values are expressed as mean ± SD, ANOVA, Tukey-Kramer *P < 0.05, **P < 0.01.

**Table 6:** The number of macrophages in each group.

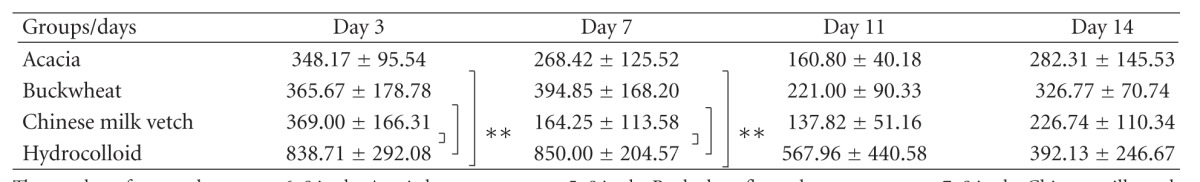

The number of macrophages: *n* = 6–8 in the Acacia honey group, *n* = 5–8 in the Buckwheat flower honey group, *n* = 7–9 in the Chinese milk vetch honey group, and *n* = 4–10 in the hydrocolloid dressing group. Values are expressed as mean ± SD, ANOVA, Tukey-Kramer **P < 0.01.

**Table 7:** The numbers of neutrophils in each group.

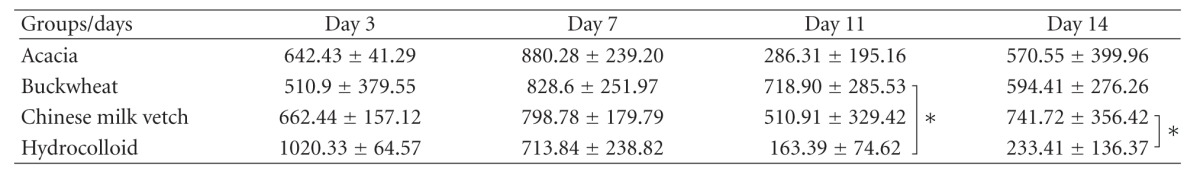

The number of neutrophils: *n* = 5–7 in the Acacia honey group, *n* = 5–8 in the Buckwheat flower honey group, *n* = 6–9 in the Chinese milk vetch honey group, and *n* = 4–7 in the hydrocolloid dressing group. Values are expressed as mean ± SD, ANOVA, Tukey-Kramer *P < 0.05.

**Table 8:** Differentiation between previous studies and present study in wound healing.

Parameter/honey	Previous studies (Various honeys)	Present study (Japanese honey)
Edema	Reduced [9, 12, 14, 20]	Not clear (macroscopic)
Debridement	Rapid autolytic [12] and less necrosis [20]	No
Wound area	Decreased [3–5, 21]	Decreased
Inflammation	Increased neutrophils [3, 11] and macrophages [4] Decreased IL- 6 [5, 8], inflammatory cell number [16], and TNF-*α* [8]	Decreased macrophages in the inflammatory phase
Reepithelialization	Promoted [4, 7, 9, 12, 14, 20, 21]	Inhibited
Angiogenesis	Stimulated [4, 12, 14]	Stimulated
Contraction	Increased [7, 20, 21]	No increase
Collagen	Increased [4, 7, 9, 21]	No increase
